# SGLT2 inhibitors and secondary polycythemia: a systematic review and meta-analysis of hematologic effects in patients with type 2 diabetes

**DOI:** 10.1007/s00228-026-04160-1

**Published:** 2026-08-13

**Authors:** Elrazi Awadelkarim Hamid Ali, Marrita Rabadi, Ruba Adel Aweer, Eihab Subahi, Elabbass A Abdelmahmuod, Awni Alshurafa, Mohammed Abdulgayoom, Mohamed Fawzi Mudarres, Usra Elshaikh, Abdulrahman Al-Mashdali, Shehab Mohamed

**Affiliations:** 1 Department of Hematology and Medical Oncology, University of Louisville, UofL Health - Brown Cancer Center , 529 S Jackson St, Louisville, KY 40202 USA; 2 College of Medicine, QU Health, Qatar University, Doha, Qatar; 3 Department of internal Medicine, University of Toledo, Toledo, OH USA; 4 Hamad Medical Corporation, Qatar metabolic institute, Doha, Qatar; 5 National Center for Cancer Care and Research, Doha, Qatar; 6 Mercy Hospital, St louis, MO USA; 7 Department of Public Health, College of Health Sciences, QU Health, Qatar University, Doha, Qatar

**Keywords:** SGLT2 inhibitors, Erythrocytosis, Polycythemia, Hemoglobin, Hematocrit, Type 2 diabetes mellitus

## Abstract

**Background:**

Sodium-glucose cotransporter 2 (SGLT2) inhibitors are widely used in the management of type 2 diabetes mellitus (T2DM) due to their survival benefits. However, these agents are associated with increases in hemoglobin (Hgb) and hematocrit (Hct), raising concern for secondary polycythemia or erythrocytosis and potential thrombotic risk. The clinical significance of these hematologic changes remains unclear.

**Methods:**

We conducted a systematic review and meta-analysis in accordance with PRISMA guidelines. PubMed, Scopus, and Embase were searched up to February 2026 for studies evaluating the effect of SGLT2 inhibitors on hematologic parameters in adults with T2DM. Patients with primary polycythemia, end-stage renal disease (ESRD), or heart failure were excluded. Primary outcomes were changes in Hgb and Hct. Secondary outcomes included the incidence of erythrocytosis/polycythemia and thrombotic events.

**Results:**

A total of 10 studies met inclusion criteria, comprising randomized and observational studies, with follow-up ranging from 8 weeks to 24 months. SGLT2 inhibitors, including empagliflozin, dapagliflozin, and canagliflozin, were consistently associated with increases in Hgb and Hct. Across three randomized controlled trials, SGLT2 inhibitors significantly increased hematocrit (3 studies, 171 patients, pooled mean difference + 2.29%, 95% CI 1.48 to 3.09) and hemoglobin (3 studies, 171 patients, pooled mean difference + 0.51 g/dL, 95% CI 0.28 to 0.74) compared with placebo. Real-world data (9,646 patients) reported an increase in polycythemia prevalence from 2.4% to 9.7%, with severe cases in 1.4% of patients. Limited data suggested a potential association with thrombotic events, with one observational study of 100 patients with JAK2-unmutated erythrocytosis reporting a 10% event rate. Hematologic changes were observed early after treatment initiation and appeared reversible following drug discontinuation.

**Conclusions:**

In patients with type 2 diabetes, ESRD, or heart failure, SGLT2 inhibitor use is associated with consistent increases in hemoglobin and hematocrit. Although these effects may contribute to cardiovascular benefit, a subset of patients may develop clinically significant erythrocytosis. Clinicians should be aware of this potential effect and monitor high-risk individuals. Larger prospective studies are needed to better define the clinical significance of these findings.

**Supplementary Information:**

The online version contains supplementary material available at https://doi.org/10.1007/s00228-026-04160-1.

## Introduction

 Sodium-glucose cotransporter 2 (SGLT2) inhibitors have become an integral element in the treatment of type 2 diabetes mellitus (T2DM), offering significant cardiovascular and renal protection in addition to glycemic control. Major adverse cardiovascular events, heart failure hospitalization, and the advancement of chronic renal disease have all been shown to decrease in large randomized controlled trials such as the CANVAS Program and the EMPA-REG OUTCOME study [1, 2]. Beyond these proven advantages, SGLT2 inhibitors consistently raise hemoglobin and hematocrit, a phenomenon that was first thought to be the result of plasma volume contraction caused by osmotic diuresis [2–4]. However, growing evidence indicates that hemoconcentration is not the only cause of this hematologic impact. Mechanistic research has shown that SGLT2 inhibition may promote erythropoiesis by increasing the synthesis of erythropoietin (EPO), most likely through enhanced renal cortical oxygenation and tubulointerstitial function restoration [3, 4]. This dual mechanism raises important questions regarding whether these hematologic changes are physiological or pathological. Clinically, elevations in hemoglobin and hematocrit are particularly significant because they can result in secondary polycythemia, a disorder linked to increased blood viscosity and an increased risk of thromboembolic events [5, 6]. Although slight increases in hematocrit may enhance oxygen delivery and possibly account for some of the cardiovascular benefits seen with SGLT2 inhibitors, excessive erythrocytosis may potentially counteract these favorable effects by increasing vascular risk. Significant erythrocytosis linked to SGLT2 inhibitor therapy has been reported in recent case reports and observational studies, indicating that in certain people, the hematologic reaction may outweigh physiological adaptation [7, 8].

Nonetheless, the clinical implications of hematologic parameter increases induced by SGLT2 inhibitors remain incompletely understood. Changes in hemoglobin and hematocrit have been reported as secondary or exploratory outcomes in the majority of randomized controlled trials, and no previous synthesis has explicitly assessed whether these increases approach thresholds consistent with secondary polycythemia. Therefore, the purpose of this study is to investigate the possible correlation between SGLT2 inhibitors and secondary polycythemia or erythrocytosis, as well as to systematically assess the impact of these drugs on hematologic markers. We aim to evaluate the extent of hemoglobin and hematocrit alterations and determine if these changes may have clinically significant consequences by combining data from observational research and randomized controlled trials.

## Methodology

This systematic review and meta-analysis was conducted in accordance with the Preferred Reporting Items for Systematic Reviews and Meta-Analyses (PRISMA) guidelines [9]. The review protocol has been submitted for registration in the International Prospective Register of Systematic Reviews (PROSPERO), and registration is currently pending. This review was also organized using the PICO framework, wherein the population is patients with type 2 diabetes mellitus who were on SGLT2 inhibitors. Patients with ESRD, heart failure, non-diabetic patients, patients on diuretics or medications causing erythrocytosis such as testosterone were excluded. SGLT2 inhibitor medication was the intervention of interest, whereas comparison groups received conventional care or a placebo. Changes in hematologic indices, such as hemoglobin and hematocrit, as well as the development of secondary polycythemia or erythrocytosis, were the outcomes of interest.

For consistency throughout this review, the term “erythrocytosis” was used preferentially over “secondary polycythemia,” as it reflects current hematologic nomenclature. Erythrocytosis was defined as an elevation in hemoglobin and/or hematocrit above sex-specific thresholds (hemoglobin > 16.5 g/dL or hematocrit > 49% in men, and hemoglobin > 16.0 g/dL or hematocrit > 48% in women). Erythrocytosis was further classified as relative or absolute, with absolute erythrocytosis subdivided into primary and secondary forms. Nevertheless, the term “secondary polycythemia” was used selectively when discussing historical literature, providing explanatory context, or when maintaining consistency with terminology reported in the original publications [6, 10, 11].

### Search strategy

Using several online databases, PubMed, Scopus, and Embase, a thorough literature search was conducted without time constraints from the database’s establishment until February 2026. Keywords, Emtree terms, and Medical Subject Headings (MeSH) terms were combined in the search strategy as follows:

(“SGLT2 inhibitors” OR “empagliflozin” OR “dapagliflozin” OR “canagliflozin”) AND (“haematocrit” OR “haemoglobin” OR “polycythemia” OR “erythrocytosis”) AND (diabetes mellites type 2 OR type 2 diabetes OR T2DM OR Non-Insulin Dependent Diabetes Mellitus).

### Eligibility criteria

Studies that presented original patient data assessing the impact of SGLT2 inhibitors on hematologic markers in individuals with type 2 diabetes mellitus were considered. Randomized controlled trials, cohort studies, case-control studies, and case reports or case series were among the study types that qualified.

Studies were included if they reported hemoglobin and/or hematocrit outcomes in adults (≥ 18 years) with type 2 diabetes mellitus receiving SGLT2 inhibitors. Papers were excluded if they involved the use of SGLT2 inhibitors for chronic kidney disease (CKD) or heart failure (HF), preclinical and animal research, and abstracts from conferences, reviews, and editorials.

### Outcomes

The study’s primary outcomes were the post-treatment variations in hemoglobin (g/dL) and hematocrit (%) between the SGLT2 inhibitor and comparator groups, which were quantitatively combined in the meta-analysis. The occurrence of erythrocytosis or secondary polycythemia, as well as other reported hematologic adverse events, were secondary outcomes. However, because of the limited and inconsistent reporting across trials, these secondary outcomes were not pooled and were instead assessed through narrative synthesis.

### Study selection

After duplicate records were eliminated, study selection was carried out using Rayyan, a systematic review management tool [12]. Title and abstract screening was the first step in the screening process, which was followed by a full-text examination of studies that can be eligible. Two reviewers evaluated each record separately, each blind to the other’s conclusions. Pre-established inclusion and exclusion criteria were used to analyze the articles. A third reviewer was consulted if a consensus could not be established after disagreements were resolved through discussion.

### Extraction of data

Using a standardized data extraction form in Microsoft Excel, two reviewers independently extracted the data. Any disagreements between reviewers were resolved by discussion or, if required, by consulting a third reviewer. Study characteristics (author, year, country, and study design), patient demographics (sample size, age, and gender distribution), intervention specifics (type of SGLT2 inhibitor, dosage, and duration), and outcomes of interest (baseline and post-treatment hemoglobin and hematocrit values) were all extracted. The length of follow-up and reported cases of erythrocytosis or secondary polycythemia were also noted, when available.

### Quality assessment

The methodological quality and risk of bias of the included studies were assessed using validated appraisal tools appropriate for each study design to ensure a consistent and methodologically rigorous evaluation of the evidence. Randomized controlled trials were evaluated using the Cochrane Risk of Bias 2 (RoB 2) tool because it is the recommended instrument for assessing bias arising from randomization, deviations from intended interventions, missing outcome data, outcome measurement, and selective reporting [13] (Table 1). Observational cohort studies were assessed using the Newcastle–Ottawa Scale (NOS), which evaluates participant selection, comparability of study groups, and outcome assessment [14] (Table 2). Case reports, case series, and analytical cross-sectional studies were appraised using the corresponding Joanna Briggs Institute (JBI) Critical Appraisal Checklists, which are specifically designed to evaluate methodological quality and reporting completeness for these study designs [15] (Tables 3 and 4). Two reviewers independently performed all quality assessments, with disagreements resolved through discussion or consultation with a third reviewer when necessary.

**Table 1:** Newcastle–Ottawa Scale (NOS) assessment of cohort studies

Author, year	Selection (max 4)	Comparability (max 2)	Outcome/Exposure (max 3)	Total
Gosmanov, 2024 [19]	4/4	2/2	2/3*	8/9
Hirose, 2016 [20]	3/4	1/2	1/3	5/9
Schwarz, 2024 [21]	3/4	0/2	3/3	6/9

The Newcastle–Ottawa Scale (NOS) was used to assess the methodological quality of non-randomized studies. The scale evaluates three domains: Selection of study groups, Comparability of groups, and Outcome/Exposure ascertainment, with a maximum score of 9 points. Higher scores indicate better methodological quality

*Asterisk indicates a study-specific note to be clarified in the main text or footnote, if applicable

**Table 2:** Risk of Bias 2 (ROB 2) assessment of randomized studies

Author, year	D1	D2	D3	D4	D5	Overall
Thiele, 2021 [18]	Low risk	Low risk	Some concerns	Low risk	Some concerns	Some concerns
Ghanim, 2020 [16]	Low risk	Low risk	Low risk	Low risk	Low risk	Low risk
van Raalte, 2019 [22]	Low risk	Low risk	Low risk	Low risk	Some concerns	Low risk
Mazer, 2020 [4]	Low risk	Low risk	Low risk	Low risk	Some concerns	Low risk

The Risk of Bias 2 (ROB 2) tool was used to assess randomized trials across five domains: D1, bias arising from the randomization process; D2, bias due to deviations from intended interventions; D3, bias due to missing outcome data; D4, bias in measurement of the outcome; and **D5**, bias in selection of the reported result. The overall risk of bias judgment was derived from these domain-level assessments

**Table 3:** JBI critical appraisal of case reports

Author, year	Q1	Q2	Q3	Q4	Q5	Q6	Q7	Q8	Overall
Gangat, 2023 [23]	Y	Y	Y	Unclear	Y	Y	Partial	Y	Include
Gupta, 2020 [8]	Y	Y	Y	Unclear	Y	Y	Y	Y	Include

The Joanna Briggs Institute (JBI) critical appraisal checklist for case reports was used to assess reporting quality. Q1 = patient demographics; Q2 = patient history and timeline; Q3 = clinical condition at presentation; Q4 = diagnostic tests and results; Q5 = intervention or treatment procedure; Q6 = post-intervention clinical condition; Q7 = adverse or unanticipated events; Q8 = takeaway lessons. Overall appraisal indicates whether the study was included, excluded, or required further information

**Table 4:** JBI critical appraisal of analytical cross-sectional study

Author, year	Q1	Q2	Q3	Q4	Q5	Q6	Q7	Q8	Overall
Hoca & Kalayci, 2025 [24]	Y	Y	Y	Y	Unclear	Unclear	Y	Y	Include

The Joanna Briggs Institute (JBI) critical appraisal checklist for analytical cross-sectional studies was used to assess methodological quality. Q1 = inclusion criteria; Q2 = study subjects and setting; Q3 = exposure measurement; Q4 = condition measurement using objective criteria; Q5 = confounding factors identified; Q6 = strategies to address confounding; Q7 = outcome measurement; Q8 = statistical analysis. Overall appraisal indicates whether the study was included, excluded, or required further information

### Statistical analysis

A preliminary, exploratory meta-analysis was conducted using randomized trials reporting hematocrit (Hct) and/or hemoglobin (Hgb) at baseline and follow-up after treatment with an SGLT2-inhibitor. The effect measure for each study was the between-group difference in change from baseline (mean difference MD) between the SGLT2-inhibitor and placebo groups. Change-from-baseline (or baseline-adjusted) estimates were preferentially used because they account for potential baseline imbalances between treatment groups and provide a more appropriate measure of treatment effect than post-treatment values alone, particularly in small randomized trials. Two studies reported baseline-adjusted between-group difference and corresponding 95% confidence interval (CI) directly [4, 16], whereas for one study the MD was calculated as the difference between groups in the mean change from baseline using arm-level summary data. All effect estimates were converted to common units before pooling, such as HGB reported in g/L converted to g/dL [17].

Hematocrit and Hemoglobin changes were analyzed separately. Study‑level MDs were pooled using a random‑effects model with restricted maximum likelihood (REML) estimation. Given the small number of included studies, CI were calculated using the Knapp–Hartung adjustment. Between‑study heterogeneity was assessed using the I² statistic, the between‑study variance (τ²), and Cochran’s Q test. A two‑sided *p* < 0.05 was considered statistically significant.

Due to the small sample size, where only three randomized trials met the criteria for quantitative synthesis, subgroup analysis, meta‑regression, and formal evaluation of publication bias were not performed. The included studies differed in follow‑up duration (3 to 6 months) and in the SGLT2 inhibitor evaluated (empagliflozin in two studies [4, 17], dapagliflozin in one [16]). These differences are acknowledged as potential sources of clinical heterogeneity and estimates should be interpreted cautiously. Analyses were performed in Stata SE 19.5 (StataCorp LLC, College Station, TX).

## Results

### Study characteristics

**Fig. 1:**
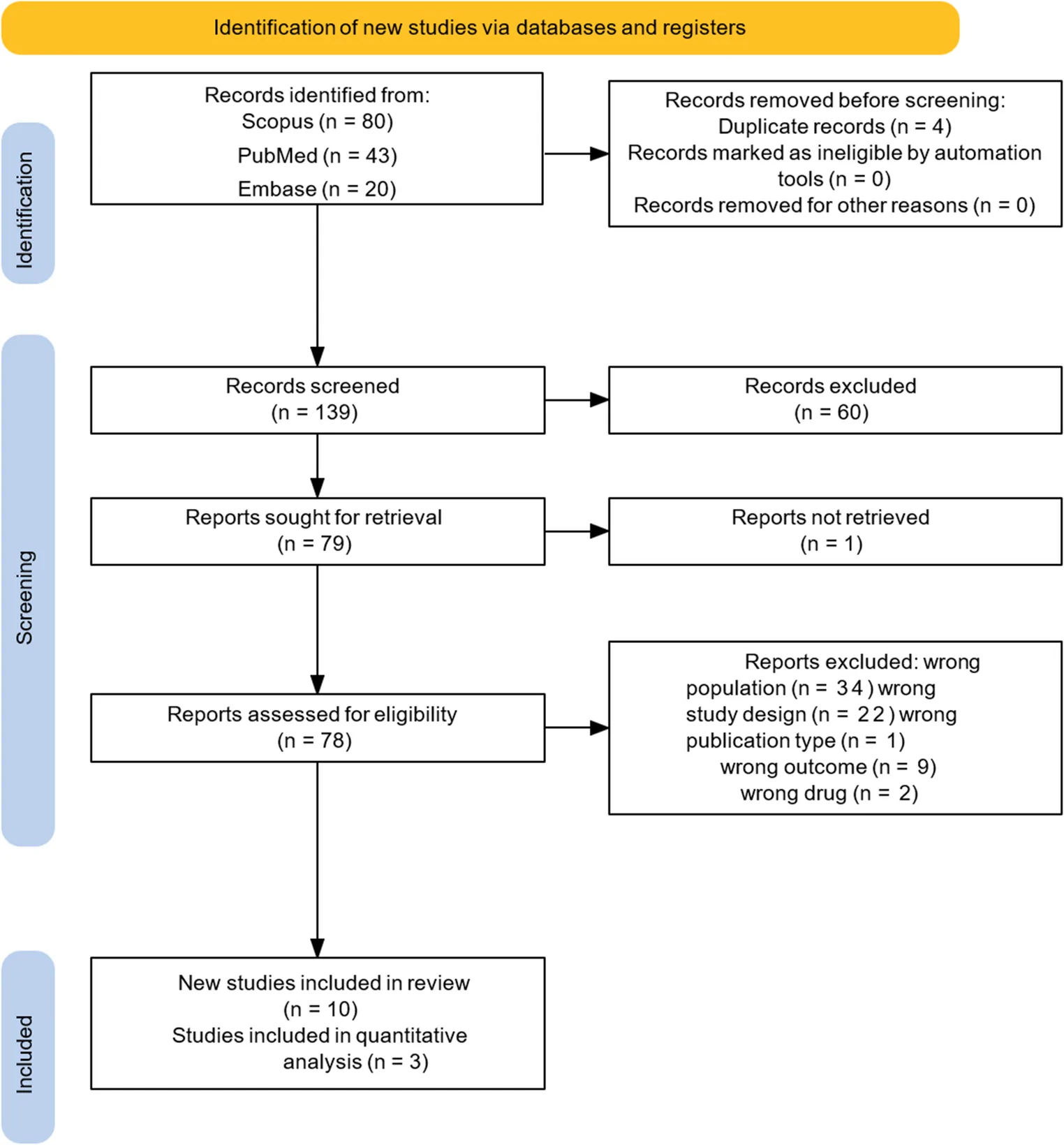
PRISMA flow diagram of the study selection process for studies evaluating SGLT2 inhibitors and erythrocytosis/polycythemia in patients with type 2 diabetes mellitus

A total of 10 studies met the inclusion criteria, comprising three randomized controlled trials, three observational cohort studies, one analytical cross-sectional study, two case reports, and one case series (Fig. 1). Follow-up durations ranged from 8 weeks to 24 months, with the case reports extending to 4 years. The most commonly evaluated SGLT2 inhibitors were empagliflozin and dapagliflozin, although canagliflozin, sotagliflozin, and tofogliflozin were also represented. Clinical outcome data were limited; only one observational study reported thrombotic events, documenting an incidence of approximately 10% (both venous and arterial events). These findings are summarized in the narrative synthesis (Sect.  3.4 and Table 5).

**Table 5:** Summary of studies included in the narrative synthesis evaluating hematologic outcomes following SGLT2 inhibitor therapy

Author, Year	Sample Size	Follow-up	SGLT2 Agent(s)	Hemoglobin (Hgb)	Hematocrit (Hct)	Polycythemia erythrocytosis / (%)	Thrombotic Events	*P*-value
Hoca & Kalayci, 2025 [24]	216 (130 users, 86 non-users)	Cross-sectional	Empagliflozin (92), Dapagliflozin (38)	14.44 vs. 13.76 g/dL	NR	NR	NR	< 0.001
Gosmanov et al., 2024 [19]	53,971	12 months	Primarily Empagliflozin	NR	+ 1.5% increase	1.4% overall (↑ to 4.0% with TRT)	NR	NR
Gangat et al., 2023 [23]	100	Median 24 months	Empagliflozin, Dapagliflozin, Canagliflozin	+ 2.5 g/dL (median)	NR	NA	10% (venous + arterial)	Dose-dependent (*p* = 0.01–0.02)*
Thiele et al., 2021 [18]	44 randomized	3 months	Empagliflozin	↑ from 136 → 142 g/L	↑ from 40.6% → 42.2%	NR	NR	0.008
Gupta et al., 2020 [8]	2 (case series)	2–4 years	Canagliflozin, Empagliflozin	↑ up to 18–19.7 g/dL	↑ up to 54–56%	NA	NR	NR
Ghanim et al., 2020 [16]	47 (24 vs. 23)	12 weeks	Dapagliflozin	↑ 13.4 → 13.9 g/dL	↑ 41.3% → 43.5%	NR	NR	0.02
van Raalte, 2019 [22]	1,575	52 weeks	Sotagliflozin	NR	↑ 41.9% → ~44%	NR	NR	NR
Hirose, 2016 [20]	20 (17 completed)	8 weeks	Tofogliflozin	NR	↑ 40.3% → 42.6%	NR	NR	< 0.001
Schwarz, 2024 [21]	9,646	~ 8.6 months	Empagliflozin (69.7%), Dapagliflozin (30.3%)	NR	↑ 41.6% → 43.7%	2.4% → 9.7% (severe: 1.4%)	NR	< 0.001
Mazer et al., 2020 [4]	90 (82 analyzed)	6 months	NR	+ 4.96 g/L	+ 2.34%	NR	NR	0.0156

*Hgb* hemoglobin, *Hct* hematocrit, *SGLT2* sodium-glucose cotransporter 2, *T2DM* type 2 diabetes mellitus, *NR* not reported, *NA* not applicable, *TRT* testosterone replacement therapy

### Effect of SGLT2 inhibitors on hematocrit

**Fig. 2:**
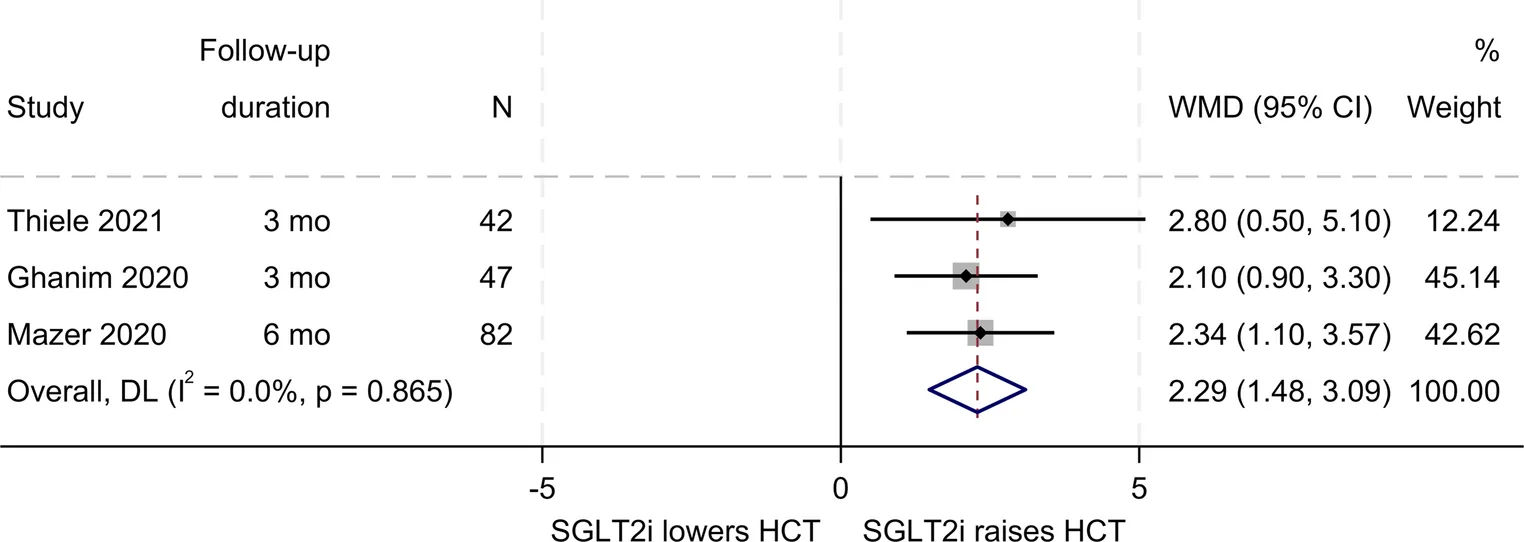
Forest plot showing the effect of SGLT2 inhibitors compared to placebo on Hct

Three RCTs comprising a total of 171 patients reported change-from-baseline hematocrit data [4, 16, 18]. Compared with placebo, SGLT2-inhibitor therapy was associated with a statistically significant increase in hematocrit, with a pooled mean difference of 2.29% (95% CI 1.48–3.09, *p* = 0.005) (Fig. 2). Although no statistical heterogeneity was detected (I² = 0%), the inclusion of only three randomized trials substantially limits the power of heterogeneity statistics to detect true between-study differences. Therefore, the absence of statistical heterogeneity should not be interpreted as evidence that clinical or methodological heterogeneity does not exist, and these findings should be interpreted cautiously (I² = 0.0%, τ² = 0, Cochran’s Q = 0.29, *p* = 0.865).

### Effect of SGLT2 inhibitors on hemoglobin

**Fig. 3:**
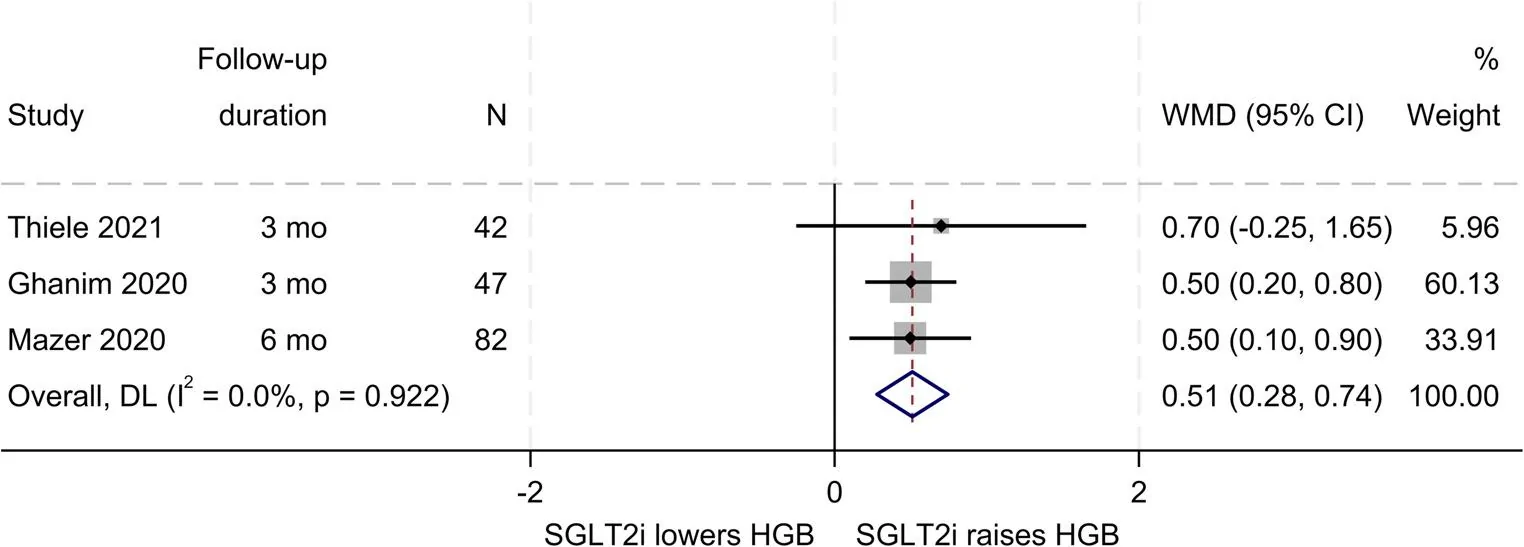
Forest plot showing the effect of SGLT2 inhibitors compared to placebo on Hgb

The same three RCTs (*n* = 171) reported change-from-baseline for HGB [4, 16, 18]. Compared with placebo, SGLT2 inhibitor therapy was associated with a statistically significant increase in HGB, with a pooled mean difference of 0.51 g/dL (95% CI 0.28–0.74, *p* = 0.004) (Fig. 3). As with hematocrit, only three randomized trials contributed to the hemoglobin analysis (I² = 0.0%, τ² = 0, Cochran’s Q = 0.16, *p* = 0.922) therefore, despite an I² of 0%, heterogeneity statistics should be interpreted cautiously.

### Narrative synthesis

Several outcomes were narratively synthesized since they could not be quantitatively pooled due to heterogeneity in study design, outcome reporting, and follow-up duration (Table 5). Although the degree of change varied among study designs and patient demographics, all included studies generally showed increases in hemoglobin and/or hematocrit after SGLT2 inhibitor medication [4, 7, 8, 16, 19–24]. To elaborate, reported hemoglobin increases ranged from modest changes (e.g., 13.4 to 13.9 g/dL in short-term studies [16]) to more pronounced elevations (up to 18–19.7 g/dL in long-term observations [8]). Similarly, hematocrit levels increased by approximately 1.5–2.5% on average, with some studies reporting higher rises over extended follow-up [4, 7, 16, 18–20].

Randomized controlled trials demonstrated statistically significant increases in hemoglobin and hematocrit within 3–6 months of treatment [16, 18]. For instance, Mazer et al. reported a significant adjusted increase in both Hgb (+ 4.96 g/L, *p* = 0.0156) and Hct (+ 2.34%, *p* = 0.0003) over 6 months [4]. Similarly, longer-term observational cohorts, including Schwarz et al. and van Raalte et al., demonstrated sustained increases in hematocrit that were observed over periods ranging from several months to one year, with mean increases of approximately 1.5–2.1% [20, 25]. Similarly, cross-sectional analysis by Hoca and Kalayci (2025) reported that hemoglobin levels were significantly higher in SGLT2 users than in non-users, measuring 14.44 g/dL compared to 13.76 g/dL, *p* < 0.001 [26], while the large population-based study by Gosmanov et al. (*n* = 53,971) found that hematocrit increased by about 1.5% over 12 months after starting therapy [19].

Several studies also reported clinically significant erythrocytosis with SGLT2 inhibitor use. Schwarz et al. showed a significant increase in erythrocytosis rates from 2.4% before treatment to 9.7% after treatment [20]. Severe polycythemia was seen in 1.4% of patients. Similarly, small cohort and case reports, such as those by Gangat et al. and Gupta et al., showed rises in hemoglobin levels of up to 2.5 g/dL or more [7, 8].

Clinical outcome data were limited. Some patients needed therapeutic phlebotomy before hematologic parameters returned to normal after discontinuing treatment [7, 8]. A study including 100 consecutive cases of JAK2-unmutated erythrocytosis reported a 10% incidence of thrombotic events (both arterial and venous) during follow-up, raising concerns about potential clinical consequences of SGLT2-associated erythrocytosis [21]. However, given the observational design, limited sample size, and potential confounding, these findings should be interpreted cautiously and do not establish a causal relationship between SGLT2 inhibitor-associated erythrocytosis and thrombotic events. Overall, while SGLT2 inhibitors were consistently associated with increases in hemoglobin and hematocrit, the clinical significance of these hematologic changes, particularly with respect to thrombotic risk, remains uncertain and requires further prospective investigation.

### Quality and risk of bias assessment

Across the included studies, methodological quality was appraised using four tools according to study design. The three cohort studies assessed with the Newcastle–Ottawa Scale showed moderate-to-high quality, with total scores ranging from 5/9 to 8/9; Gosmanov (2024) achieved the highest score (8/9) [19], while Hirose (2016) [27] and Schwarz (2024) [20] scored 5/9 and 6/9, respectively (Table 1). Among randomized trials, ROB 2 assessment indicated an overall low risk of bias for Ghanim (2020) [16], van Raalte (2019) [25], and Mazer (2020) [4], whereas Thiele (2021) [18] was assessed as having some concerns, mainly related to missing outcome data and selection of the reported result (Table 2). The two case reports appraised with the JBI checklist were both considered suitable for inclusion [7, 8], with all major reporting domains adequately addressed except for unclear reporting of diagnostic assessment in both studies and only partial reporting of adverse events in Gangat et al. (2023) (Table 3). The analytical cross-sectional study by Hoca and Kalayci (2025) [26], also assessed using the JBI checklist, was judged suitable for inclusion, with all key domains fulfilled except for unclear identification of confounding factors and unclear reporting of strategies used to address confounding (Table 4).

## Discussion

### Principal findings

Polycythemia, or erythrocytosis, is defined as an elevation in hemoglobin and/or hematocrit above normal levels due to increased red blood cell mass. Clinically, erythrocytosis is categorized as relative or absolute. Relative erythrocytosis reflects hemoconcentration due to reduced plasma volume, whereas absolute erythrocytosis represents a true increase in erythrocyte mass. Absolute erythrocytosis is further subdivided into primary and secondary forms. Primary erythrocytosis, most commonly polycythemia vera (PV), is characterized by autonomous erythropoiesis and is typically driven by JAK2 mutations. In contrast, secondary erythrocytosis results from increased erythropoietin (EPO) production in response to hypoxia, medications, or other stimuli and is more common than PV. Differentiating between these entities remains essential given the distinct management strategies required [11, 28, 29].

The differential diagnosis of secondary erythrocytosis is broad and includes hypoxia-related conditions, renal pathology, erythropoietin-producing tumors, and pharmacologic agents. Among medications, testosterone and exogenous erythropoietin are well-established contributors. More recently, sodium-glucose cotransporter 2 (SGLT2) inhibitors have emerged as an increasingly recognized cause of medication-associated erythrocytosis, particularly in patients with diabetes mellitus [4, 11, 29].

In this systematic review, SGLT2 inhibitors consistently increased hemoglobin and hematocrit across randomized trials, observational studies, and real-world cohorts. While these increases were generally modest, clinically significant erythrocytosis appeared confined to a susceptible subset of patients. These findings are consistent with large cardiovascular outcome trials, where hematocrit elevation represents a reproducible class effect of SGLT2 inhibition [2, 16, 18]. Importantly, the pooled findings should not be interpreted as indicating that all patients receiving SGLT2 inhibitors develop clinically significant erythrocytosis. The modest increases in hemoglobin and hematocrit observed in randomized controlled trials likely represent the expected pharmacodynamic effect of SGLT2 inhibition.

While average increases in hemoglobin (0.3–0.7 g/dL) and hematocrit (1–2%) are modest, the clinical relevance lies in the marked variability in hematologic response. Our analysis highlights that a subset of patients develops significant erythrocytosis, with reported hemoglobin levels exceeding 18–19 g/dL and polycythemia prevalence approaching 10% in real-world cohorts. This variability suggests heterogeneity in individual susceptibility and underscores the need for patient-level risk stratification. Risk appears to be influenced by baseline characteristics, including high-normal hemoglobin levels, concomitant testosterone therapy, and underlying renal disease [7, 8, 20].

Hematologic changes appear early after initiation of SGLT2 inhibitors, often within weeks to months, and may persist with continued therapy, supporting a sustained pharmacologic effect rather than a transient hemodynamic phenomenon. Importantly, the reversibility of erythrocytosis following discontinuation of SGLT2 inhibitors, as observed in several reports, further supports a potential drug-related association and helps distinguish this condition from primary myeloproliferative disorders.

### Mechanisms underlying SGLT2-induced erythrocytosis

From a mechanistic perspective, the erythropoietic effect of SGLT2 inhibitors extends beyond simple hemoconcentration. While early hypotheses emphasized plasma volume contraction secondary to osmotic diuresis, this mechanism alone does not explain the sustained increases observed. Current evidence supports a biologically plausible model in which SGLT2 inhibition reduces renal metabolic stress, improves cortical oxygenation, and restores erythropoietin-producing cell function. This leads to increased erythropoietin secretion and stimulation of erythropoiesis. Additionally, emerging data suggest modulation of iron metabolism pathways, including reductions in hepcidin and improved iron availability, further facilitating erythropoiesis. This supports a shift from a purely hemodynamic explanation toward a true erythropoietic model of action. These mechanisms parallel pathways described in anemia of chronic disease, where hepcidin dysregulation and impaired erythropoiesis play central roles, highlighting the complex interplay between renal physiology, inflammation, and red cell production [4, 23, 24, 30, 31].

The erythropoietic effects of SGLT2 inhibitors have been demonstrated not only in diabetes but also in chronic kidney disease (CKD) and heart failure populations, supporting a class effect independent of glycemic status. However, inclusion of these populations introduces potential confounding related to volume status, comorbidities, and concomitant therapies, reinforcing the rationale for our diabetes-focused analysis [22–24].

A critical clinical question is whether SGLT2-associated erythrocytosis translates into increased thrombotic risk. Large randomized trials have not demonstrated a consistent increase in thromboembolic events, and hematocrit elevation has been associated with improved cardiovascular outcomes in some analyses. However, smaller studies and real-world cohorts suggest that thrombotic risk may be elevated in patients who develop marked erythrocytosis, particularly in the presence of additional risk factors. Although data remain limited and heterogeneous, the observed signal warrants careful monitoring, especially in high-risk individuals [7, 19]. In addition, because the available evidence is derived predominantly from observational studies and case reports, which are susceptible to confounding and selection bias, these findings should not be interpreted as establishing a causal relationship between SGLT2 inhibitor-associated erythrocytosis and thrombotic events.

### Clinical implications

From a clinical perspective, mild increases in hemoglobin and hematocrit are expected with SGLT2 inhibitor therapy and are generally benign. However, further evaluation is warranted when elevations exceed normal thresholds, are progressive, or are associated with symptoms. Clinicians should systematically exclude secondary causes and consider hematology referral when appropriate [6, 11, 28, 29].

Overall, while SGLT2 inhibitors confer substantial cardiovascular and renal benefits, clinicians should recognize erythrocytosis as a potential pharmacodynamic effect. Recognition of this phenomenon is essential to avoid misclassification as primary polycythemia and to guide appropriate evaluation without unnecessary discontinuation of therapy. Management should be individualized, balancing the degree of hematologic change against the well-established benefits of treatment.

### Strengths

A major strength of this review is its focused evaluation of patients with diabetes mellitus, deliberately excluding populations with chronic kidney disease, heart failure, and diuretic exposure, thereby reducing confounding related to volume status and comorbid conditions. This approach allows a clearer assessment of the direct hematologic effects of SGLT2 inhibitors.

Importantly, this study integrates evidence from randomized trials, observational cohorts, and case reports into a unified synthesis, bridging the gap between controlled trial findings and real-world clinical practice. While prior literature has described modest increases in hemoglobin and hematocrit, our analysis highlights clinically relevant variability, including a subset of patients at risk for marked erythrocytosis, thereby expanding current understanding of SGLT2-associated hematologic effects.

Finally, by combining clinical data with mechanistic insights and meta-analytic findings, this review provides a structured platform for future research, including the need for prospective studies, standardized definitions, and improved risk stratification strategies.

### Limitations

This review has several limitations. First, the available literature was heterogeneous in study design, patient populations, follow-up duration, and SGLT2 inhibitors evaluated, and only three randomized controlled trials were eligible for quantitative synthesis. This precluded subgroup analyses, meta-regression, and formal assessment of publication bias. Second, evidence regarding clinically significant erythrocytosis and thrombotic events was derived primarily from observational studies and case reports, limiting causal inference. Finally, despite efforts to minimize confounding, residual bias cannot be entirely excluded, particularly related to baseline renal function, smoking status, testosterone exposure, dehydration, and unrecognized hypoxic conditions.

## Conclusion

SGLT2 inhibitors were associated with statistically significant increases in hematocrit (pooled mean difference 2.29%) and hemoglobin (pooled mean difference 0.51 g/dL), findings that were consistently supported by the narrative evidence across randomized trials, observational studies, and case reports. Although these hematologic changes are generally modest and may reflect the known erythropoietic effects of SGLT2 inhibitors, a subset of patients may develop clinically significant erythrocytosis requiring closer monitoring or intervention. Current evidence regarding thrombotic risk remains limited and is derived predominantly from observational studies and case reports, precluding definitive conclusions regarding causality. Clinicians should remain aware of this potential adverse effect, particularly in patients with additional risk factors for erythrocytosis. Further large, prospective studies are needed to better define the incidence, predictors, and long-term clinical significance of SGLT2 inhibitor-associated erythrocytosis.

## Supplementary Information

Supplementary Material 1.

## Data Availability

The data in this systematic review are generated from pre-exisiting published articles.
