# Supplementary material for: SGLT2 inhibitors and secondary polycythemia: a systematic review and meta-analysis of hematologic effects in patients with type 2 diabetes
Source: Eur J Clin Pharmacol. 2026 Aug 13;82(9):232. doi: 10.1007/s00228-026-04160-1 (PMC13469376; doi:10.1007/s00228-026-04160-1)
Supplement: Supplementary file 1 [file 228_2026_4160_MOESM1_ESM.docx]

**Search Strategy: Feb 1^st^, 2026**

**PubMed:**

**268 results**

TITLE-ABS-KEY

("SGLT2 inhibitors" OR "empagliflozin" OR "dapagliflozin" OR "canagliflozin") AND ("haematocrit" OR "haemoglobin" OR "polycythemia" OR "erythrocytosis") AND (diabetes mellitus type 2 OR type 2 diabetes OR T2DM OR Non Insulin Dependent Diabetes Mellitus).

**Scopus**

**134 results**

TITLE-ABS-KEY

("SGLT2 inhibitors" OR "empagliflozin" OR "dapagliflozin" OR "canagliflozin") AND ("haematocrit" OR "haemoglobin" OR "polycythemia" OR "erythrocytosis") AND (diabetes mellitus type 2 OR type 2 diabetes OR T2DM OR Non Insulin Dependent Diabetes Mellitus).

**Embase**

**346 Results**

TITLE-ABS-KEY

("SGLT2 inhibitors" OR "empagliflozin" OR "dapagliflozin" OR "canagliflozin") AND ("haematocrit" OR "haemoglobin" OR "polycythemia" OR "erythrocytosis") AND (diabetes mellitus type 2 OR type 2 diabetes OR T2DM OR Non Insulin Dependent Diabetes Mellitus).
